# A new method for assessment of nickel-titanium endodontic instrument surface roughness using field emission scanning electronic microscope

**DOI:** 10.1186/s12903-020-01233-0

**Published:** 2020-08-31

**Authors:** Khoa Van Pham, Canh Quang Vo

**Affiliations:** 1 Department of Operative Dentistry and Endodontics, Faculty of Odonto-Stomatology, University of Medicine and Pharmacy at Ho Chi Minh City, 652 Nguyen Trai Street, Ward 11, District 5, Ho Chi Minh City, Vietnam; 2 Faculty of Medicine, Vietnam National University Ho Chi Minh City, Ho Chi Minh City, Vietnam

**Keywords:** Endodontic, FE-SEM, Nickel-titanium, Reciprocating, Surface roughness

## Abstract

**Background:**

To introduce a new method for measurement of surface roughness of the endodontic instrument, before and after instrumentation, using the Field Emission Scanning Electronic Microscope (FE-SEM) combined with the ImageJ software.

**Methods:**

Twenty J-shape resin blocks were divided into two groups, ten blocks of each group. Simulated root canal inside the resin block was 16 mm length, 60^0^ angle of curvature, and radius of 4.5 mm. Ten WaveOne Gold Primary and 10 Reciproc Blue R25 instruments were used for root canal instrumentation. The instruments were scanned before and after instrumentation with special molds made to ensure the same areas at the point located 3 mm from the tips of the instruments using the FE-SEM. These scanned images were analyzed using the ImageJ. The arithmetical mean roughness (R_a_), root mean square roughness (R_q_), and the average distance between the highest peak and lowest valley in each sampling length (R_z_) were calculated by ImageJ for quantitative analyses. The paired-t test was performed to analyze the data using the SPSS 22.0 at the significance of .05.

**Results:**

Almost all surface roughness values were decreased. However, these decreases were not statistically significant (*P* > .05).

**Conclusions:**

The FE-SEM combined with the ImageJ was the reliable and appropriate modality for measurement surface roughness of instruments.

## Background

The root canal preparation not only is one of the most important phases in endodontics, but also is decisive for the success of the obturation step and thanks to that, is important for the whole root canal therapy [1]. Advancements in technology led to the coming out into the market of the nickel-titanium (NiTi) endodontic instruments using its own integrated techniques that provide safer, easier and faster ways to prepare the root canal systems with better maintain the original shape and fewer iatrogenic errors [1]. The producers incessantly innovate for their own products to reduce the time needed, steps in utilization, exhaustion of clinicians, and therefore, increasing the success rate in the clinical situations for these instruments [2]. In efforts to reach those goals, the single-file instrument systems were produced with two modes of rotary: continuous and reciprocating rotary [2]. Since the first reciprocating single-file system has born, this system is unceasing improved in cross-sectional design and material. Recently, the WaveOne Gold (WOG) (Dentsply Sirona, Maillefer, Ballaigues, Switzerland) and Reciproc Blue (RB) (VDW, Munich, Germany) were introduced with many special characteristics in designs and heat treatment processes [3, 4]. Both of systems were reciprocating single-file and single-use systems. The WaveOne Gold is created using proprietary thermal processing obtained by heating the NiTi file and then cooling it slowly; different from production of M-Wire, a pre-manufacturing thermal processing, which is attributed to improvement instrument’s elasticity [5]. The Reciproc Blue results from creative thermal processing that produce an instrument with a blue surface. As previously described, the manufacturers of both WaveOne Gold and Reciproc Blue instruments claim that thermal processing improves the flexibility and cyclic fatigue resistance of the NiTi instruments [6].

One of the most major drawbacks for all kind of endodontic instruments was the instrument fracture. Metals making endodontic instruments can be broken down following two mechanisms: brittle or ductile [7]. Typically, an initial crack on the metal surface will cause the instrument’s fracture [7]. Crack usually initiates from the defect on the material surface [8]. There is a relationship between the surface topography and the fracture mechanism of rotary NiTi instruments [9]. Recently, there are few studies focusing on the surface roughness of the instrument for evaluation on this value before and after root canal instrumentation [6, 10–13]. Although the atomic force microscope has capable of three-dimension (3D) image reconstruction and surface roughness measurement, there are still many certain shortcomings of this device and the non-contact optical profilometer seems the most suitable modality for investigation and measurement of the surface roughness of the endodontic instrument for the time being [12, 14]. A fundamental equipment, previously supposed that not suitable for 3D surface topography investigation, proving the auspicious candidate for this task, is the field emission scanning electronic microscope (FE-SEM) [15]. This study introduces a new method for measurement of surface roughness of the endodontic instrument, before and after resin block root canal instrumentation, using the FE-SEM combining with the ImageJ software (NIH, Bethesda, MD, USA).

## Materials and methods

Based on the pilot study, sample size calculation was performed using G*Power 3.1 software (Heinrich Heine University, Dusseldorf, Germany) by selecting the t-test, Means: Difference between two dependent means (matched pairs). The alpha-type error of 0.05, and the beta power of 0.95. The calculation showed that the sample size for each group must be a minimum of 10 instruments. Thus, ten WOG Primary and ten RB R25 instruments were included in the present study.

An acrylonitrile butadiene styrene (ABS) mold was created using computerized numerically controlled machine for keeping the endodontic instrument at the right position after root canal instrumentation. Polydimethylsiloxane (PDMS) (Sylgard 184, Dow Corning Corp., USA) was used for taking the impression of the instrument before instrumentation inside the ABS mold for each instrument, with the mark on the shaft was above. ABS mold with the instrument and PDMS impression was inserted into a vacuum extractor (Diener Electronic, Germany) to remove the bubbles inside the material and then dried on a dryer at 60^0^ C in 3 h. The ABS mold was then fixed into the vacuum chamber’s gear of the FE-SEM.

Twenty J-shape endo-training clear resin blocks (Dentsply Sirona, Maillefer, Ballaigues, Switzerland) were divided into two groups, ten blocks of each group. Simulated root canal inside the resin block was 16 mm length, 60^0^ angle of curvature, and radius of 4.5 mm, according to the Pruett’s method [16]. All root canal instrumentations were performed by a trained operator. The initial glide path was conducted by using the ISO 10 manual K-file (Dentsply Sirona, Maillefer, Ballaigues, Switzerland) until the file was loose inside the resin canal. X-Smart IQ handpiece (Dentsply Sirona, Maillefer, Ballaigues, Switzerland) and accompanied iPad Mini were used with programs in the software for root canal preparation. All reciprocating nickel-titanium instruments used for the present study were examined for any manufacturer defects under the stereomicroscope Axio Scope A1 (Carl Zeiss, Germany). Instruments without defects were included in the present study.

First, group 1 was enlarged the glide path using the WaveOne Gold Glider (Dentsply Sirona, Maillefer, Ballaigues, Switzerland) and group 2 was enlarged the glide path using the R-Pilot (VDW, Munich, Germany) until the instrument reached to the working length. Root canal was irrigated with 3% sodium hypochlorite solution (Canal Pro, Coltene Whaledent, Altstätten, Switzerland). The group 1 was then prepared using the WaveOne Gold Primary and group 2 was prepared using the Reciproc Blue R25 until the instruments reached the working length using the gentle in-and-out pecking motion to simulate clinical situations. Root canal was irrigated with 3% sodium hypochlorite solution. Every reciprocating instrument was used for only one resin canal. After root canal preparation, the surface of the instrument was cleaned by moisture gauze, then the instrument was ultrasonic cleaned in alcohol solution and then dried using gauze.

**Fig. 1:**
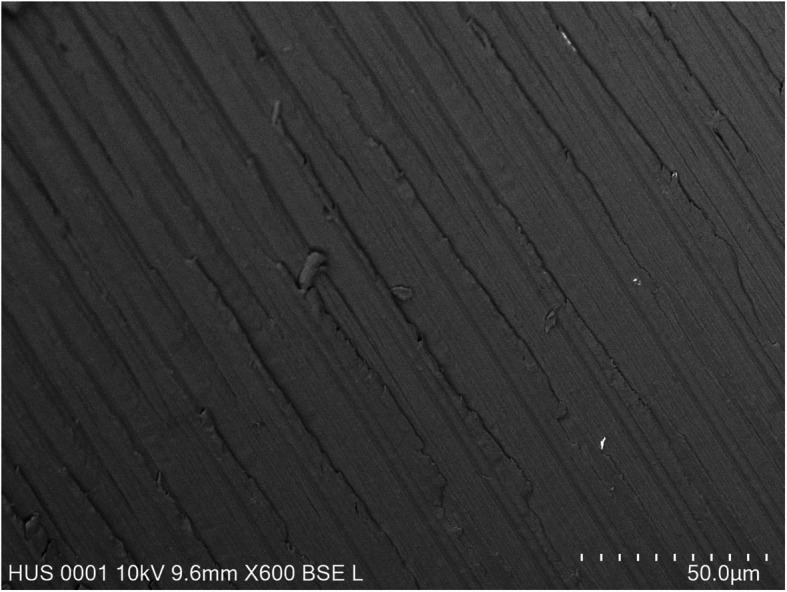
FE-SEM Image of an intact WaveOne Gold Primary Flute

**Fig. 2:**
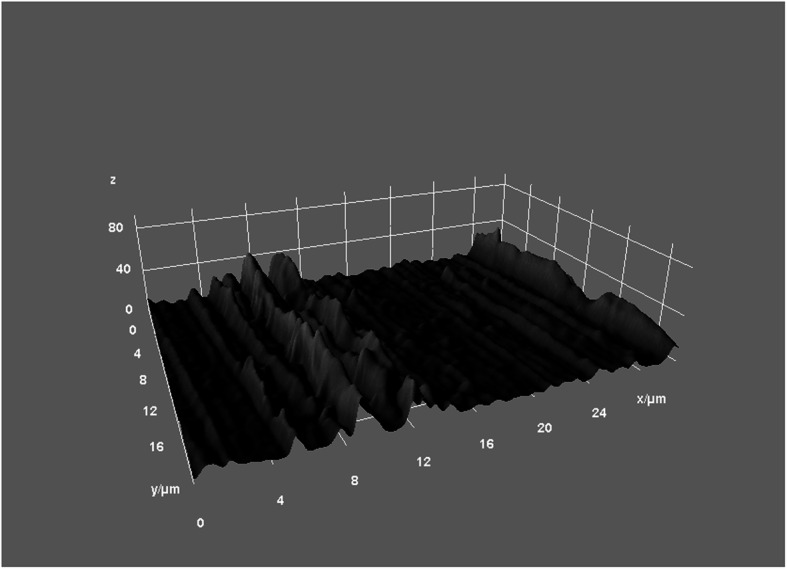
3-D Image of an intact WaveOne Gold Primary Flute

**Fig. 3:**
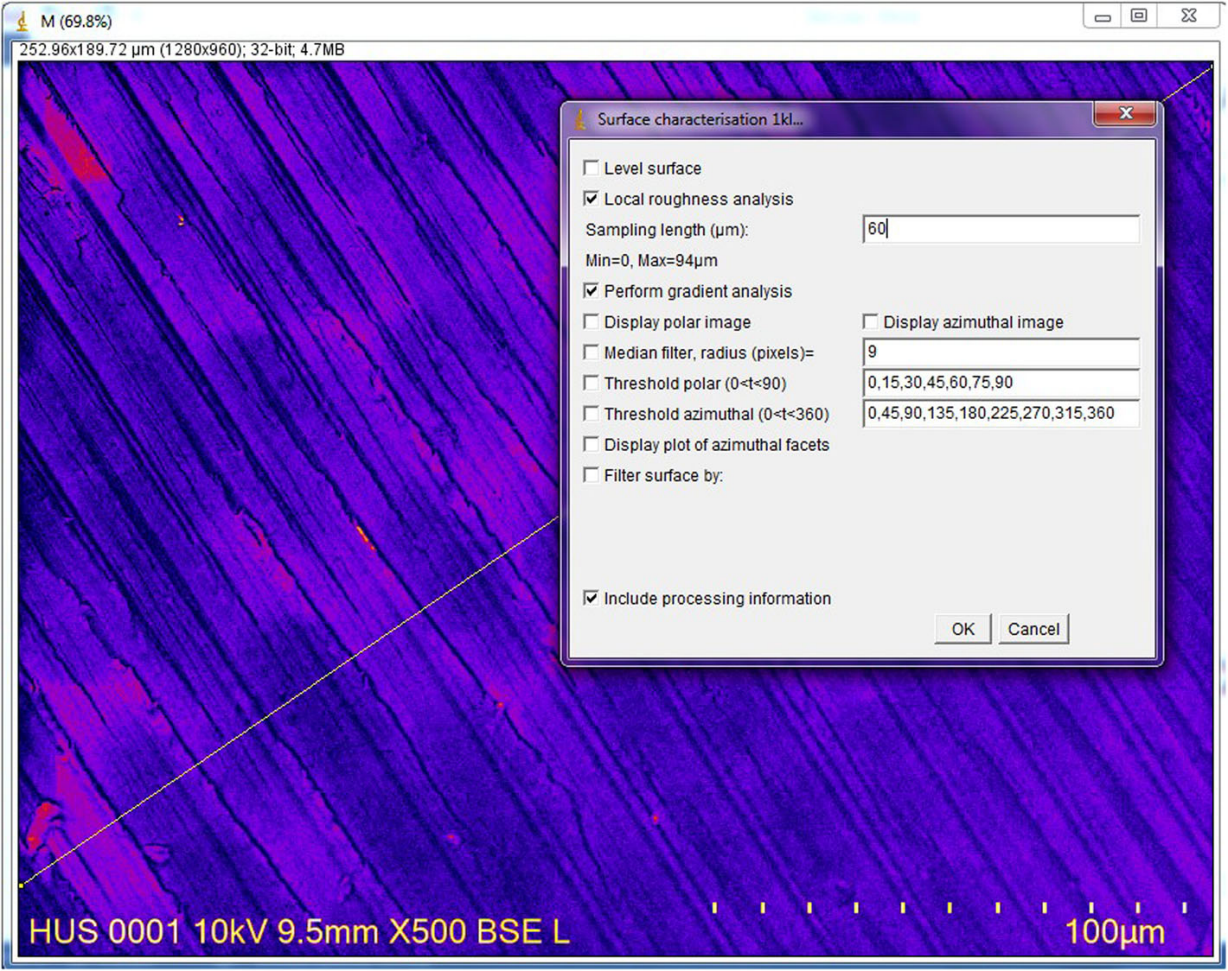
Parameters chosen on the ImageJ interface at the last step of analysis

Ten WOG Primary (lot no. 1493642) and 10 RB R25 (lot no. 280432) instruments were used for the present study. The shafts of all instruments were marked using the ISO 014 round bur (Dentsply Sirona, Maillefer, Ballaigues, Switzerland). These marks helped in reinserting the instruments into the impression after root canal instrumentation and in observing the same areas on the instruments before and after instrumentation. The instruments were scanned before and after root canal instrumentation by using the Field Emission Scanning Electronic Microscope (FE-SEM, NOVA NanoSEM 450, FEI, UNSW, Sydney, Australia). The surface of the areas located 3 mm from the tip of the instrument were evaluated using the modification from the method of Ferreira et al. [12]. Reference points were the marks on the instrument shaft. The scanning areas were reached, first by moving the gear of the vacuum chamber from the mark toward the tip until the tip can be observed. Then, the gear was moved 3 mm back from the tip, and at this point, the cutting blade and the adjacent flute were scanned and taken pictures with the dimensions of 150 μm × 150 μm (Figs. 1 and 2). These images were analyzed using the ImageJ 1.52 (NIH, Bethesda, MD, USA). The arithmetical mean roughness (R_a_), root mean square roughness (R_q_), and the average distance between the highest peak and lowest valley in each sampling length (R_z_) were calculated by ImageJ for quantitative analyses (Fig. 3).

Shapiro-Wilk test was used first to test the normality of the data. The Student *t* test was performed to analyze the data using the SPSS 22.0 (IBM-SPSS Inc., Armonk, NY, USA) software.

## Results

The mean and standard deviations of the R_a_, R_q,_ and R_z_ values were displayed in the Table 1. All these values of group 1 decreased after root canal instrumentation, however, these decreases were not statistically significant (*P* > .05), except for the R_z_ value at the flute area (*P <* .05). For the group 2, at the cutting blade area, R_q_ and R_z_ values increased and R_a_ value decreased after root canal instrumentation, however, these differences were not statistically significant (*P >* .05). At the flute area, all surface roughness values decreased after root canal instrumentation, however, these decreases were not statistically significant (*P* > .05), except for the R_q_ value (*P <* .05).

**Table 1:** Mean and Standard Deviations of R_a_, R_q_, R_z_ parameters before and after instrumentation of experimental instruments (μm)

	WOG Primary				RB R25			
	Cutting blade		Flute		Cutting blade		Flute	
Variable	Before	After	Before	After	Before	After	Before	After
R_a_	10.294 ± 4.548	9.254 ± 3.318	11.107 ± 3.269	9.324 ± 2.596	7.454 ± 2.039	7.453 ± 2.014	8.337 ± 1.798	6.532 ± 1.266
R_q_	13.402 ± 5.181	12.521 ± 4.172	14.510 ± 3.874	12.079 ± 3.428	10.054 ± 2.464	10.548 ± 3.144	10.905 ± 2.425^c^	9.116 ± 1.934^d^
R_z_	125.239 ± 22.670	112.889 ± 30.062	133.965 ± 16.878^a^	105.560 ± 24.745^b^	85.046 ± 19.477	97.521 ± 36.344	91.225 ± 23.670	83.691 ± 27.808

Different superscript letters indicated statistically significant differences at 5% level

## Discussion

The traditional SEM was widely used on evaluation the surface area of the endodontic instrument after root canal preparation. However, this modality created two-dimensional images that could not be processed for quantitative surface data [17].

The atomic force microscope (AFM) was a valuable modality in evaluation of the surface topographical characteristics of the nickel-titanium instruments [10, 11]. One of the most important disadvantages of the AFM was that the scanning area was so small, approximately 1 μm × 1 μm, 5 μm × 5 μm or 20 μm × 20 μm. The atomic force microscope (AFM) could only read on a perfect square, flat and rigid surface area. However, it was hard to find such an area that on the endodontic instrument. In the contact mode, the tip of the stylus could damage the surface topographical features [12]. The AFM spent considerable time to take the measurement. Although the AFM could offer the 3D images, it’s so small scanning area made the repeated measurement on the same area of the endodontic instrument at different times became so difficult [12].

Recently, the non-contact profilometry had been proposed by Ferreira et al. for evaluation of the endodontic NiTi instrument surface [12]. This modality could extract the data for any irregular surfaces, including rough, stepped, smooth, angle, flat, or curved, erasing the requirement of complex sample preparation with the high accuracy, at any magnification, and in a quick time. The scanning area in this technique was relatively broad, at 210 μm × 210 μm. Especially, with the special procedure, the method could make the repeatable, reproducible, and precise measurements on the same areas of the instrument surface at different times [6, 12]. However, the technique could not interpret exact results when measuring features in very slope angles or discontinuities [18]. The imperfections of the optical components caused aberrations that would affect the optical resolution and the precision of measurements [18]. The surface roughness was generally overestimated by optical instrumentation, and multiple scattering was the cause. The results from the optical measurements differed significantly from other measurement techniques [18]. The optical instrument relied on the reflection of an electromagnetic wave; therefore, the good reflective surface was the requirement of observed material. One of the most important shortcomings of the optical profilometer was that it could not perform roughness measurement on the fracture surfaces [19]. The software-dependent situation of all modern software-integrated optical profilometers made the modification of data become difficult and impossible for these devices, and they could not work any longer whenever the data processing software was broken down.

High resolution FE-SEM used the electron instead of light. The endodontic nickel-titanium instrument was made from electrically conductive material; therefore, it could be directly made measurements in the FE-SEM, required not any sample prepared procedure. The FE-SEM used for the present study reached the resolution to 1.4 nm. One of the most important advantages of utilization of the FE-SEM for the present study was the combination of the data from the FE-SEM and the free ImageJ 1.52 software in interpreting the results of surface roughness parameters and the 3-D images of the scanning surface. The precision and reliability of the method using the ImageJ software for measuring surface roughness parameters and reconstructing the 3-D images of the scanning surface from the data of FE-SEM were confirmed by other previous studies [15, 20, 21]. In the present study, the surface roughness parameters and the reconstruction of the 3-D images were successfully performed by the free independent ImageJ 1.52 software using the data of the images obtained from the NOVA FE-SEM. This opened the new direction for quantitative analysis and evaluation on the surface of the endodontic instruments before and after root canal instrumentation. The scanning area was 150 μm × 150 μm suitable for evaluation the cutting blade and especially the adjacent flute because this dimension was smaller than the distance between two cutting blades at the point of 3 mm from the tip of the instrument. The PDMS material using in forming the impression for instrument was the material for the vacuum environment of the FE-SEM chamber. Working temperature of this material ranged from -40 °C to 150 °C and the dimensional change of the material was inconsiderable in the vacuum chamber [22]. Along with the precision of the ABS mold created by the CNC, the gear of the FE-SEM chamber revealed the ability of the system in ensuring the same area of the surface instrument was evaluated before and after root canal instrumentation.

Surface roughness parameters could be measured by many modalities as above discussion and using many parameters. R_a_, R_q_, R_z_ were most popular parameters used for endodontic instrument surface. R_a_ value could be inversely proportional to the number of cycles to fatigue fracture of the endodontic instruments [23].

Although the extracted human tooth could be a better object for study because it could simulate more appropriate clinical treatment situation, the resin endo-training block could still be the standardized and acceptable model for experimental study. The hardness value, length, diameter, curvature in terms of angle and radius of the resin block were standardized to ensure there was not any sample bias in the study compared with that of the extracted human tooth. However, the hardness of the resin was lower than that of human dentin, that led to the less wear of the instrument surface after root canal instrumentation [24]. One other disadvantage of the resin block was the material could be melt when subject to the heat resulted from the root canal preparation and the material could accumulate in flutes of the instrument and this could cause the tip of the instrument be stuck in the root canal and lead to the fracture of instrument [13]. For some certain purposes, the resin blocks seemed unreliable, such as in cutting efficiency or apical transportation evaluation [2, 25].

Creating and maintaining a smooth, reproducible, and secured glide path was an important characteristic of proper root canal preparation [26]. The glide path played an important role in all phases of the root canal preparation, preventing the procedural severe errors [27]. Although certain new reciprocating nickel-titanium instrument using the advanced thermal process could reach full root canal working length without previous secured glide path [4], the glide path preparation still was an important step in root canal preparation [27].

The results of the present study revealed that surface changes, even the smallest ones, could be detected using this new method using FE-SEM at high accuracy. Although, almost surface roughness values of the WOG group were not significantly reduced, these values might be resulted from the effectiveness of the glide path preparation using the WOG Glider in the previous step of root canal preparation. The R_z_ values of the cutting blades were significant decreased after root canal preparation in the WOG group however, this parameter was local characteristic more than an entire representative surface roughness value. These results were like those of the previous study [6]. The R_a_ and R_q_ values of the flute in the RB group were significantly reduced after root canal preparation. This revealed that the RB instruments were subjected to the friction more than the WOG instruments did. This could be the result from the differences between the design, dimension, and material of the R-Pilot instrument for the glide path creating and the RB instrument for the root canal preparation. These results did not agree with those in the previous study [6], in that, all surface roughness values of RB group were increased after root canal instrumentation. These differences might come from the differences in the study design and other conditions such as resin versus human extracted teeth, one resin canal versus four human mesial root canals, glide path preparation with K-file or with mechanical instruments. The reduction of the surface roughness values at specific areas revealed that there was almost not any wear on the surface of these areas of the instruments. Contact areas of the instruments were subjected to light loads and experienced fine polishing that was governed by the ordinarily dislocation plasticity [28].

The present study used one instrument for only one canal at the room temperature and resin canal that could affect the results of the study.

The surface roughness of the nickel-titanium instruments is an important characteristic and relates to the fracture mechanism of the instruments. Measurement of these values using the common device as the FE-SEM is affordable for almost researchers.

## Conclusions

The FE-SEM combined with the ImageJ software was the reliable and appropriate modality for evaluation of the endodontic instrument surface roughness. The surface roughness values of the WOG Primary and RB R25 were not statistically changed after the first utilization in glide path created resin root canals.

## Data Availability

The datasets used and/or analyzed during the current study are available from the corresponding author on reasonable request.

## Abbreviations

3-D: Three dimension
ABS: Acrylonitrile butadiene styrene
AFM: Atomic force microscope
FE-SEM: Field emission scanning electronic microscope
NiTi: Nickel-Titanium
PDMS: Polydimethylsiloxane
RB: Reciproc Blue
WOG: WaveOne Gold
